# TRIM37 Mediates Chemoresistance and Maintenance of Stemness in Pancreatic Cancer Cells *via* Ubiquitination of PTEN and Activation of the AKT–GSK-3β–β-Catenin Signaling Pathway

**DOI:** 10.3389/fonc.2020.554787

**Published:** 2020-10-16

**Authors:** Shiyu Chen, Zhiwei He, Changhao Zhu, Yanqing Liu, Lin Li, Lu Deng, Jun Wang, Chao Yu, Chengyi Sun

**Affiliations:** ^1^School of Basic Medical Sciences, Guizhou Medical University, Guiyang, China; ^2^Department of Hepatobiliary Surgery, The Affiliated Hospital of Guizhou Medical University, Guiyang, China; ^3^Guizhou Provincial Institute of Hepatobiliary, Pancreatic and Splenic Diseases, Guiyang, China; ^4^Key Laboratory of Liver, Gallbladder, Pancreas and Spleen of Guizhou Medical University, Guiyang, China; ^5^Reproductive Medicine Center, The Affiliated Hospital of Guizhou Medical University, Guiyang, China

**Keywords:** TRIM37, pancreatic cancer, phosphatase and tensin homolog, stemness, chemoresistance

## Abstract

**Purpose:**

The tripartite motif-containing family member TRIM37 is involved in a number of important biological and pathological processes, and it has recently been shown to be an essential regulator of protein ubiquitination and a contributor to tumorigenesis. We previously showed that TRIM37 is overexpressed in and promotes the proliferation and invasion of pancreatic cancer (PC).

**Methods:**

Sphere formation, flow cytometric, qRT-PCR, western blot, colony formation, EdU incorporation, mouse xenograft model, TUNEL and IHC assays were performed to detect the role of TRIM37 in stemness and chemoresistance of PC *in vitro* and *in vivo*. Bioinformatics analysis and dual-luciferase reporter assays were used to determine which intracellular pathways might mediate the effects of TRIM37 in PC cells. Immunofluorescent(IF) staining, co-immunoprecipitation(CO-IP), protein stability and ubiquitination assays were performed to investigate the relationship between TRIM37 and PTEN.

**Results:**

TRIM37 modulates the ubiquitination and degradation of the tumor suppressor phosphatase and tensin homolog (PTEN), which negatively regulates the AKT–GSK-3β–β-catenin signaling pathway, thereby sustaining aberrant activation of PC cells. High expression of TRIM37 combined with low expression of PTEN correlates with poor survival of PC patients.

**Conclusions:**

Collectively, our results suggest that inhibition of the TRIM37–AKT–GSK-3β–β-catenin axis may be a promising strategy for treatment of PC.

## Introduction

Pancreatic cancer (PC) is the most lethal and aggressive cancer and has a dismal prognosis, as illustrated by a 5-year overall survival rate of about 6% and a median survival time from diagnosis of about 6 months (1–3). The current treatment strategy for PC is surgical resection followed by a comprehensive regimen of chemotherapy and radiotherapy, which leads to a modest improvement in overall survival (4, 5). However, most PC is refractory to treatment and patients generally relapse, ultimately succumbing to the disease. Extensive work over the past decade has identified tumor cells with stem cell-like traits (cancer stem cells, CSCs) that are believed to be responsible for the initiation, progression, and metastasis of a variety of cancers, including PC (6–10). Therefore, there is an urgent need to understand the molecular basis of both chemoresistance and stemness of PC to provide novel therapeutic targets.

The large tripartite motif-containing (TRIM) family of proteins is characterized by the presence of a canonical N-terminal TRIM domain, which is highly conserved and is composed of a Really Interesting New Gene (RING) finger domain, B-boxes, and a Coiled-Coil domain. TRIM family proteins participate in a variety of cellular processes, including proliferation, transcriptional regulation, differentiation, and tumorigenesis (11–14). TRIM37, which is encoded by a gene in the 17q23 chromosomal region, has been shown to play important roles in cancer progression (11–14), at least in part through the activities of its RING finger domain, which functions as an E3 ubiquitin ligase and promotes protein degradation *via* the ubiquitin–proteasome system. TRIM37 activity is known to modulate the expression of multiple genes, including tumor suppressors, in several types of cancers (15, 16). TRIM37 enhances the invasion and metastasis of many cancers, including gastric cancer, glioma, and colorectal cancer, *via* the epithelial–mesenchymal transition (17–19). We previously showed that TRIM37 promotes the migration of PC and hepatocellular carcinoma cells (20, 21).

The protein kinase B (AKT) –glycogen synthase kinase-3β (GSK-3β) –β-catenin signaling pathway has been reported to play an important role in cancer stemness and chemoresistance (22–25). This pathway is regulated in part through the activity of phosphatase and tensin homolog (PTEN), a key tumor suppressor that negatively regulates phosphatidylinositol 3-kinase (PI3K)–AKT signaling. PTEN is one of the most frequently mutated genes in human cancer, and its aberrant regulation has been extensively studied in many tumor types, including PC (26), colorectal cancer (27), breast cancer (28), and lung cancer (29).

In the present study, we examined the involvement of TRIM37 in the growth of PC and show that it confers chemoresistance and stemness *via* ubiquitination and degradation of PTEN, leading to activation of the AKT–GSK-3β–β-catenin signaling pathway. In addition, we identify a significant correlation between the prognosis of PC patients and tumor expression levels of TRIM37 and PTEN, confirming the clinical relevance and potential therapeutic utility of our results.

## Materials and Methods

### Human Tissue Samples

The PC tissue microarray was obtained from Shanghai Outdo Biotech (Shanghai, China). Surgical specimens of PC and adjacent normal pancreatic tissue were obtained from 110 patients (61 men, 49 women) who underwent surgical resection for PC from January 2014 to December 2017 (**Supplementary Table 1**). All PC specimens were histologically classified as pancreatic adenocarcinomas. This study was approved by the Human Research Ethics Committee at the Affiliated Hospital of Guizhou Medical University.

### Chemicals and Cell Culture

All antibodies were purchased from Cell Signaling Technology (Beverly, MA, USA) and Abcam (Cambridge, MA, USA). PANC-1 and MIA PaCa-2 cell lines were purchased from the American Type Culture Collection (ATCC; Manassas, VA, USA). Both cell lines were previously authenticated by ATCC by short tandem repeat typing. PANC-1 and MIA PaCa-2 cells were grown in DMEM medium (Gibco, Waltham, MA, USA) supplemented with 10% fetal bovine serum (Gibco), 100 U/ml penicillin G (Beyotime Biotechnology, Wuhan, China), and 100 μg/ml streptomycin (Beyotime Biotechnology) at 37°C in a humidified atmosphere containing 5% CO_2_.

### RNA Preparation and qRT-PCR

RNA was extracted from tissues and cells using TRIzol reagent (Invitrogen, Carlsbad, CA, USA) and PrimeScript RT reagent (TaKaRa, Dalian, China) was used for reverse transcription to obtain cDNA samples. The expression status of specific genes and GAPDH were determined by quantitative real-time RT-PCR using a Quantitative Real-Time PCR system (Bio-Rad, Hercules, CA, USA) with primers listed in the **Supplementary Table 2**.

### Western Blot Analysis

Tumor tissues and cells were lysed in RIPA buffer (Pierce) containing protease inhibitors (Boster Biological Technology, Wuhan, China). Protein in the lysates was quantified using a BCA assay kit (Beyotime Biotechnology). Aliquots of 40 μg total protein were separated by SDS-PAGE and transferred to polyvinylidene fluoride membranes (Millipore, Temecula, CA, USA). The blots were probed using the antibodies listed in **Supplementary Table 3**.

### Lentivirus Infection

Lentiviruses carrying a TRIM37-FLAG overexpression vector or encoding short hairpin RNA (shRNA) targeting TRIM37 were purchased from GeneChem (Shanghai, China). Cells were infected with lentiviruses at a multiplicity of infection of 50. At 48 h post-transfection, expression was verified by RT-qPCR and western blot analysis.

### Sphere-Formation Assay

PC cells PANC-1 and MIA PaCa-2 (600/well) were seeded into 6-well ultra-low attachment cluster plates (Corning; Corning, NY, USA) and cultured in serum-free DMEM/F12 medium (Invitrogen) supplemented with 0.4% BSA (Sigma-Aldrich, St. Louis, MO, USA), 2% B27 (Invitrogen), 5 μg/ml insulin (Sigma-Aldrich), 20 ng/ml EGF (PeproTech, Rocky Hill, CT), and 20 ng/ml bFGF (PeproTech). Two weeks later, cell spheres were photographed and counted once weekly.

### Colony-Formation Assay

PC cells PANC-1 and MIA PaCa-2 (1×10^3^/well) were seeded into 6-well culture plates and incubated for 14 days. The cells were then fixed by addition of 4% paraformaldehyde and stained with 0.25% crystal violet. The culture plates were photographed and the colonies were enumerated.

### Flow Cytometric Analysis

PC cells PANC-1 and MIA PaCa-2 were seeded into 6-well plates, incubated for 24 h, collected, and washed with PBS. The cells were then incubated with FITC-annexin V and propidium iodide (PI) in binding buffer (BD, La Jolla, CA, USA) for 30 min in the dark at room temperature and analyzed using a FACS flow cytometer (Becton Dickinson, Franklin Lakes, NJ, USA).

### Mouse Xenograft Model

The animal experimental protocol was approved by the Institutional Animal Care and Use Committee of Guizhou Medical University. PANC-1 cells (Vector, TRIM37 overexpression, shRNA-V, and TRIM37 knockdown) were stably transfected with the firefly luciferase gene. And these PANC-1 cells were injected subcutaneously (2×10^6^/mouse) into the right axilla of 4 groups(n=5/group) of 6 weeks female BALB/c nude mice. Starting 1 week later, all of the mice were injected intraperitoneally with normal saline contained 5-Fluorouracil (5-FU) (150 mg/kg) twice a week for 45 days. And the *in vivo* bioluminescent assay was detected by the IVIS imaging system. Then the tumors were excised and volumes were calculated using the equation (Length × Width^2^)/2. Tumors were sectioned or lysed for further analysis.

### Immunofluorescence Microscopy

PC cells were incubated overnight on the slides in 6-well plates, and then fixed with 4% paraformaldehyde. The cells were then incubated with TRIM37 and PTEN antibodies (**Supplementary Table 3**) diluted in 5% bovine serum albumin at 4°C for 24 h. After washing with ice-cold PBS, the cells were incubated with the appropriate TRIM37/PTEN-conjugated second antibodies for 2 h. The cells were then washed again, stained with 4′,6-diamidino-2-phenylindole to identify cell nuclei, and analyzed using a confocal laser microscope.

### Immunohistochemical Staining

Tissue sections were incubated with antibodies (**Supplementary Table 3**) and incubated overnight at 4°C. The slides were then washed and incubated with appropriate secondary antibodies at 37°C for 0.5 h. The staining intensity and extent (positively stained area) in three randomly selected areas was evaluated independently by two of the authors (CZ and LL).

### Immunoprecipitation and Mass Spectrometry (IP-MS)

Cells were lysed and incubated with TRIM37 and PTEN antibodies (**Supplementary Table 3**) for 4 h at 4°C with rotation. The lysate mixture was then mixed with Sepharose-conjugated protein G beads (Beyotime) and incubated overnight at 4°C. The lysates were centrifugated, and the beads were collected, washed three times with ice-cold PBS, incubated with 2× loading buffer and boiled to isolate the protein. Further analysis was carried out by sodium dodecyl sulfate polyacrylamide gel electrophoresis separation and subsequently analyzed by western blot or MS.

### Dual-Luciferase Reporter Assay

PC cells were incubated overnight in 6-well plates and then transfected with 0.5 μg each of the appropriate overexpression vector, 0.5 μg of TCF-TOP/FOP promoter-luciferase reporter plasmid, and 0.01 μg of pRL-TK plasmid (internal control). Approximately 48 h after transfection, the cells were lysed and luciferase activity was measured using a Dual Luciferase Reporter Assay Kit (Promega), according to the manufacturer’s instructions.

### Statistical Analysis

All statistical analyses were performed using SPSS 22.0 software (IBM, Armonk, NY, USA). For continuous variables, the data are presented as the mean ± SD. Student’s t-test (two-tailed) was used to examine differences between two groups and one-way ANOVA was used to evaluate three or more groups. *P* < 0.05 was considered to indicate statistical significance.

## Results

### TRIM37 Promotes CSC-Like Traits and Chemoresistance of PC Cells *In Vitro*

**Figure 1:**
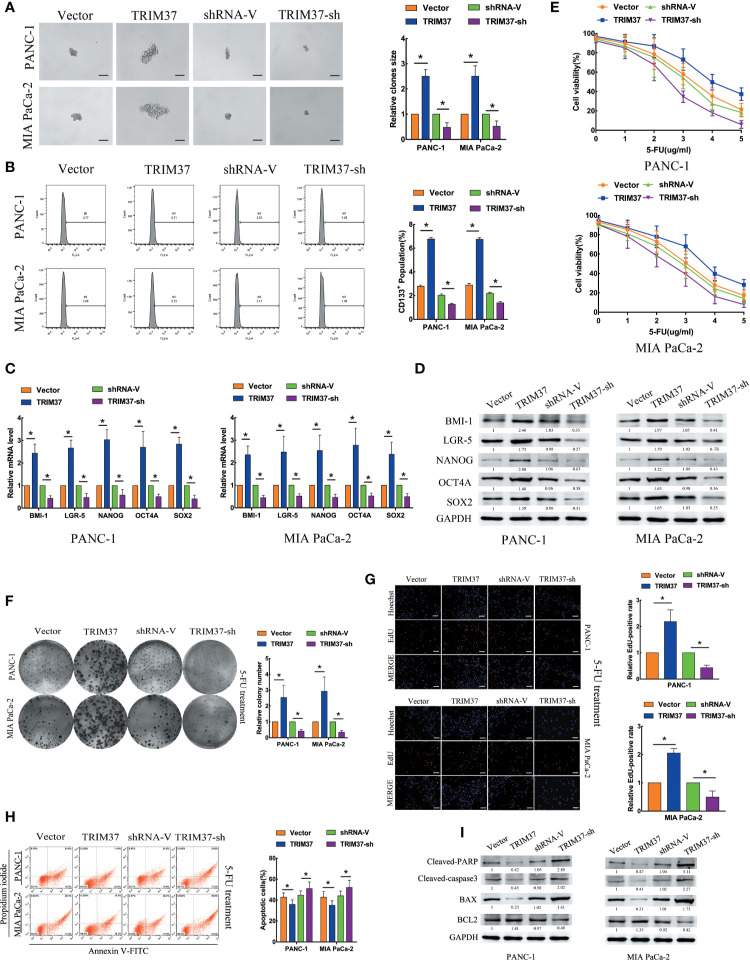
TRIM37 promotes PC stem cell-like traits and chemoresistance *in vitro*. **(A–I)** PANC-1 and MIA PaCa-2 human PC cells were infected with lentiviruses encoding TRIM37 or TRIM37-specific shRNA. Cells were analyzed for: **(A)** sphere formation, scale bar, 100 um; **(B)** expression of CD133 by flow cytometry; **(C, D)** expression of pluripotency-related molecules by qRT-PCR **(C)** and western blot analysis **(D)**; **(E–G)** 5-FU resistance by CCK-8, colony formation, and EdU assays, scale bar, 100 um; **(H)** apoptosis of PC cells by flow cytometry; **(I)** expression of cleaved PARP, cleaved caspase-3, BAX and BCL2 by western blot analysis. *(P < 0.05).

To investigate the role of TRIM37 in stemness and chemoresistance of PC, we infected two human PC cell lines, PANC-1 and MIA PaCa-2, with lentiviruses harboring either a TRIM37 overexpression vector or TRIM37-specific shRNA (**Figure S1**). We first examined sphere formation, which is a common method of identifying cells with CSC-like properties. Overexpression and silencing of TRIM37 dramatically increased and decreased, respectively, the size of spheres in both PC cell lines compared with the control cell lines (**Figure 1A**). Flow cytometric analysis of the cells indicated that TRIM37 overexpression increased the proportion of cells expressing CD133, a CSC marker, and conversely, TRIM37 silencing decreased the CD133^+^ cell population (**Figure 1B**). qRT-PCR and western blot analysis showed that cells overexpressing TRIM37 had significantly upregulated expression of the stemness markers BMI-1 (B-cell-specific moloney murine leukemia virus insertion site-1), LGR-5 (leucine rich repeat containing G protein-coupled receptor-5), NANOG (Nanog homeobox), OCT4A (POU class 5 homeobox 1), and SOX2 (SRY-box transcription factor 2), whereas TRIM37 knockdown had the opposite effect (**Figures 1C, D**). These data support the involvement of TRIM37 in maintaining the CSC-like phenotype of PC cells.

To explore the role of TRIM37 in PC cell chemoresistance, we cultured PANC-1 and MIA PaCa-2 cells with the chemotherapeutic agent 5-Fluorouracil (5-FU). TRIM37 overexpression or silencing significantly increased or decreased, respectively, the IC50 of 5-FU (**Figure 1E**). Consistent with this, TRIM37-overexpressing PC cells showed enhanced colony-forming ability (**Figure 1F**) and proliferation, as measured by EdU incorporation (**Figure 1G**), compared with control PC cells. As expected, TRIM37 silencing had the opposite effects (**Figures 1F, G**). Moreover, the rate of 5-FU-induced apoptosis was much lower for TRIM37-overexpressing PANC-1 and MIA PaCa-2 cells compared with control cells, whereas the rate was much higher for TRIM37-silenced cells (**Figure 1H**). Finally, expression of the apoptosis-related proteins cleaved poly ADP-ribose polymerase (cleaved PARP), cleaved caspase-3, and BCL2 (B-cell lymphoma 2) associated X (BAX) was significantly reduced in TRIM37-overexpressing PC cells and increased in TRIM37-silenced cells compared with control cells, whereas BCL2 had the opposite effect (**Figure 1I**). Collectively, these results suggest that TRIM37 promotes CSC reprogramming and 5-FU resistance in PC cells.

### Overexpression of TRIM37 Confers 5-FU Resistance and CSC-Like Traits on PC Cell *In Vivo*

**Figure 2:**
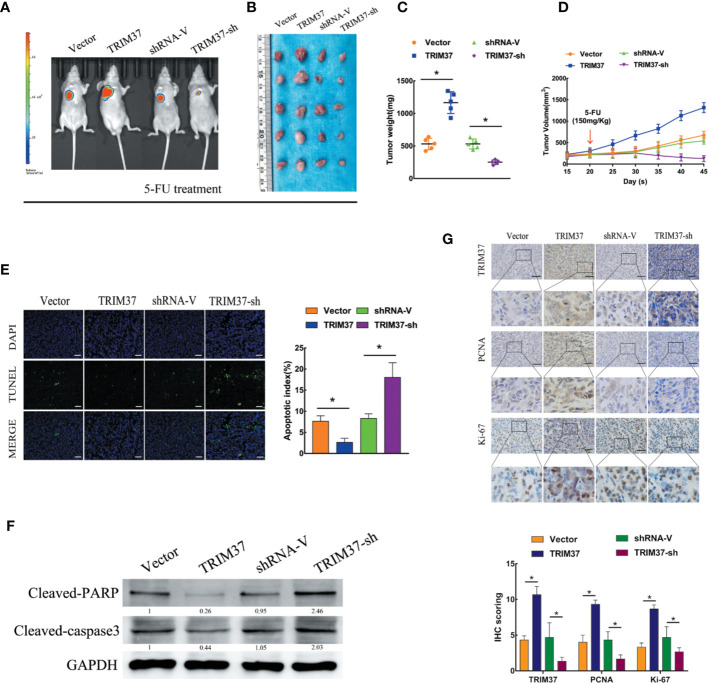
TRIM37 overexpression confers 5-FU resistance and stemness on PC cells *in vivo*. **(A–G)** Control, TRIM37-overexpressing, or TRIM37-silenced PANC-1 cells were injected subcutaneously into BALB/c nude mice, treatment with 5-FU (150 mg/kg) was started one week later, and continued twice a week. **(A)** Representative images of tumor-bearing mice. **(B)** Tumors from all mice and mean tumor weights **(C)**. **(D)** Tumor volumes on the indicated days. **(E)** TUNEL staining of excised tumor sections (*P *< 0.05), scale bar, 100 um. **(F)** Western blot analysis of cleaved PARP and cleaved caspase-3 in lysates of the indicated tumors. **(G)** IHC staining of TRIM37, PCNA, and Ki-67 in sections of the indicated tumors, scale bar, 100 um. *(P < 0.05).

To determine whether our *in vitro* findings were consistent with the functions of TRIM37 in *in vivo*, we employed a BALB/c nude mouse xenograft model. Groups of mice were subcutaneously injected with control, TRIM37-overexpressing, or TRIM37-silenced PANC-1 cells, and the mice were then treated with 5-FU (150 mg/kg) twice per week, beginning when the tumor reached 0.5 cm in diameter. We found that TRIM37 overexpression significantly increased tumor growth in 5-FU-treated mice, whereas downregulation of TRIM37 significantly inhibited tumor growth (**Figures 2A–D**). Consistent with our *in vitro* findings, the tumors formed by TRIM37-overexpressing PANC-1 cells were more resistant than control tumors to 5-FU-induced apoptosis, as measured by the proportion of TUNEL^+^ cells (**Figure 2E**), indicating that TRIM37 silencing enhanced the cytotoxic effect of 5-FU on PC cells. In addition, western blot analysis of excised tumors demonstrated that TRIM37 significantly inhibited expression of cleaved PARP and cleaved caspase-3 (**Figure 2F**), indicating reduced apoptosis, and IHC staining demonstrated enhanced PCNA and Ki-67 expression in TRIM37-overexpressing PANC-1 tumors (**Figure 2G**), consistent with increased proliferation. Finally, we found that expression of stemness markers were significantly increased in the TRIM37-overexpressing tumors compared with control tumors (**Figure S2**). The opposite effects were consistently observed with PANC-1 cells expressing TRIM37-specific shRNA (**Figure 2** and **Figure S2**). Collectively, these results demonstrate that TRIM37 contributes to the chemoresistance and stemness of PC cells *in vivo*.

### TRIM37 Overexpression Activates the AKT–GSK-3β–β-Catenin Signaling Pathway in PC

**Figure 3:**
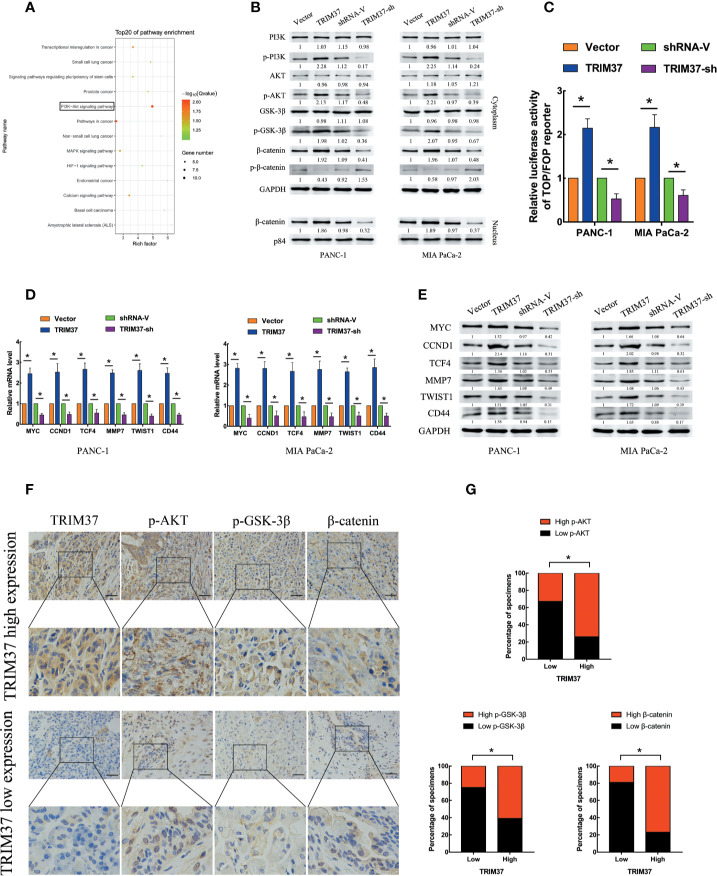
TRIM37 overexpression activates the AKT–GSK-3β–β-catenin signaling pathway in PC cells. **(A)** KEGG pathway analysis of TRIM37 expression and its correlation with PI3K–AKT signaling pathway genes in the TCGA PAAD dataset. **(B)** Western blot analysis of PI3K, p-PI3K, AKT, p-AKT, GSK-3β, p-GSK-3β, β-catenin, p-β-catenin, and nuclear β-catenin in the indicated PC cells. **(C)** TCF4/LEF1-driven luciferase reporter assay in the indicated PC cells at 48 h after transfection. Firefly luciferase activity was normalized to *Renilla* luciferase activity. **(D, E)** RT-qPCR **(D)** and western blot analysis **(E)** of expression of MYC, CCND1, TCF4, MMP7, TWIST1, and CD44 in the indicated cells. **(F, G)** IHC analysis of TRIM37, p-AKT, p-GSK-3β, and β-catenin expression in serial sections of human PC specimens, scale bar, 100 um, and TRIM37 expression level correlates positively with AKT–GSK-3β–β-catenin signaling pathway components in clinical PC specimens. *(P < 0.05).

To assess which intracellular pathways might mediate the effects of TRIM37 overexpression and silencing in PC cells, we performed Kyoto Encyclopedia of Genes and Genomes (KEGG) pathway enrichment analysis of genes in the pancreatic adenocarcinoma dataset from The Cancer Genome Atlas (TCGA). We found that the expression of components of the PI3K–AKT signaling pathway were positively correlated with TRIM37 gene expression (**Figure 3A**). Consistent with this, western blot analysis of PANC-1 and MIA PaCa-2 cells revealed that the expression of the active, phosphorylated (p) forms of PI3K, AKT, and GSK-3β, and β-catenin levels were significantly increased in TRIM37-overexpressing but inhibited in TRIM37-silenced cells compared with control cells (**Figure 3B**). Nuclear β-catenin levels were also increased by TRIM37 overexpression and decreased by TRIM37 silencing (**Figure 3B**). Indeed, dual-luciferase reporter assays indicated that TRIM37 overexpression or silencing significantly enhanced or suppressed, respectively, transcription of the β-catenin target gene TCF4/LEF1 (**Figure 3C**). Furthermore, mRNA and protein levels of the well-characterized AKT–GSK-3β–β-catenin target genes MYC, CCND1, TCF4, MMP7, TWIST1, and CD44 were upregulated in TRIM37-overexpressing cells and downregulated in TRIM37-silenced cells (**Figures 3D, E****)**. In addition, IHC of 30 cases human PC specimens demonstrated that TRIM37 expression correlated positively with the expression of p-AKT, p-GSK-3β, and β-catenin (**Figures 3F, G**).

### TRIM37 Drives 5-FU Resistance of PC Cells Through the AKT–GSK-3β–β-Catenin Pathway

**Figure 4:**
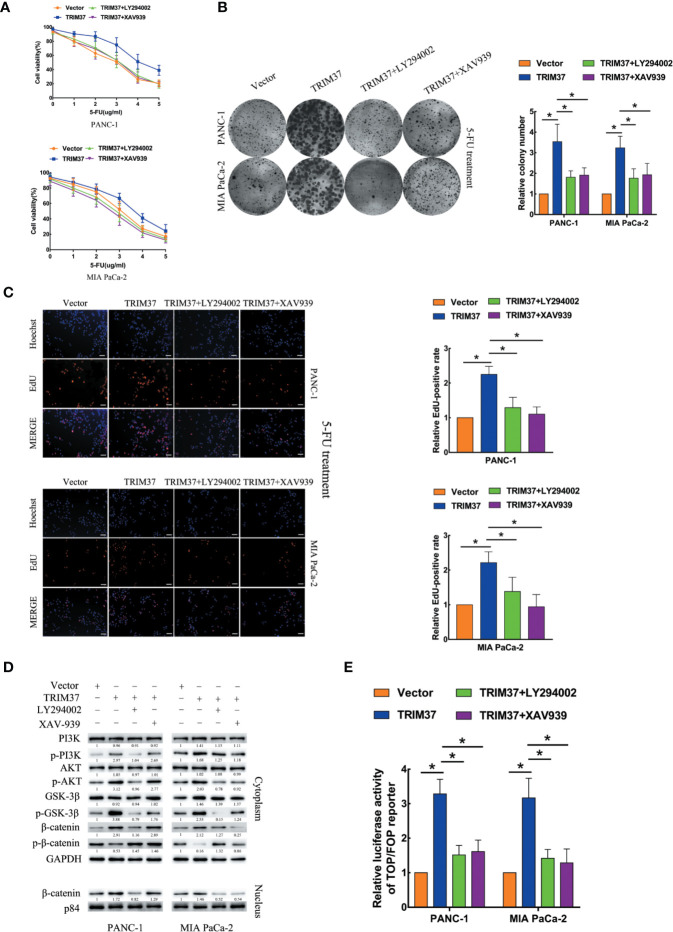
TRIM37 drives 5-FU resistance in PC cells through the AKT–GSK-3β–β-catenin signaling pathway. **(A–C)** CCK-8 **(A)**, colony formation **(B)**, and EdU, scale bar, 100 um **(C)** assays of the indicated cells treated with 5-FU, with/without LY294002 (PI3K inhibitor) and XAV-939 (β-catenin inhibitor). **(D)** Western blot analysis of PI3K, p-PI3K, AKT, p-AKT, GSK-3β, p-GSK-3β, β-catenin, p-β-catenin, and nuclear β-catenin in the indicated cells incubated with LY294002 or XAV-939. **(E)** TCF4/LEF1-driven luciferase reporter assay of the indicated cells incubated with LY294002 or XAV-939. *(P < 0.05).

We next investigated the contribution of the AKT–GSK-3β–β-catenin pathway to the chemoresistance-promoting effect of TRIM37 in PC cells by performing rescue experiments with LY294002 and XAV-939, which are small molecule inhibitors of PI3K and β-catenin, respectively. We found that both of the inhibitors blocked TRIM37-mediated 5-FU resistance in PC cells, as measured by cell viability (IC50), colony formation, and cell proliferation (EdU) assays (**Figures 4A–C**). Moreover, treatment of cells with either of the two inhibitors reversed the effects of TRIM37 on the expression levels of p-PI3K, p-AKT, p-GSK-3β, and cytoplasmic/nuclear β-catenin (**Figure 4D**) and on TCF4/LEF1-driven luciferase reporter activity (**Figure 4E**). Taken together, these results demonstrate that TRIM37-induced resistance of PC cells to 5-FU is mediated, at least in part, through the AKT–GSK-3β–β-catenin pathway.

### TRIM37 Mediates the Ubiquitination and Degradation of PTEN in PC Cells

Given that TRIM37 has E3 ubiquitin ligase activity, we hypothesized that one mechanism by which TRIM37 may function in PC cells is through ubiquitination-promoted degradation of key proteins. To identify potential TRIM37-binding proteins, we immunoprecipitated TRIM37 from PC cells and performed IP-MS on the immunoprecipitated material (**Supplementary Table 4**). This analysis identified PTEN as a major binding partner of TRIM37, which was of particular interest because PTEN is known to regulate the AKT–GSK-3β–β-catenin pathway.

**Figure 5:**
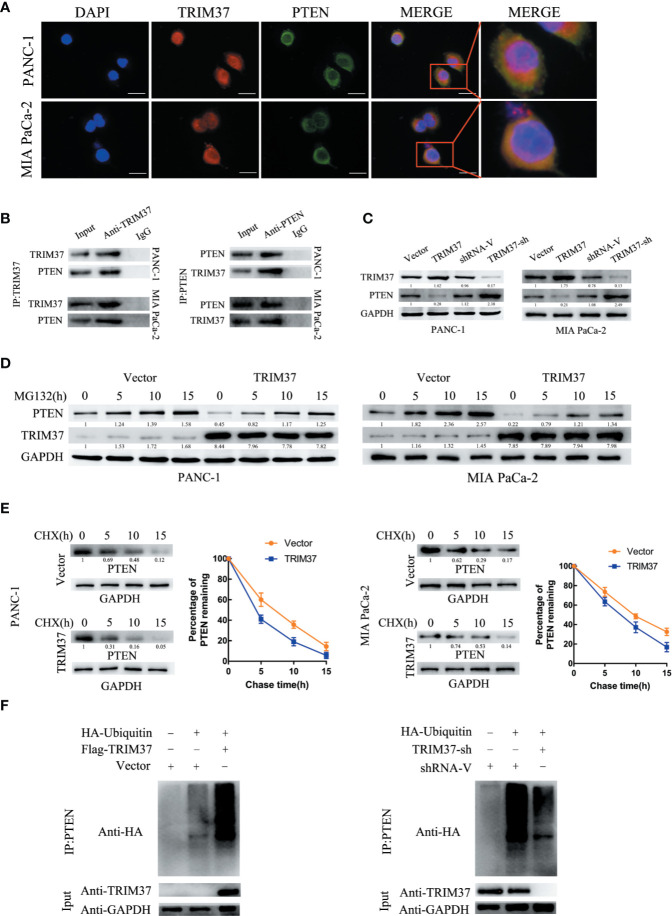
TRIM37 downregulates the expression of PTEN in PC cells *via* ubiquitination and degradation. **(A)** Confocal microscopy of TRIM37 and PTEN in the indicated cells, scale bar, 50 um. **(B)** Immunoprecipitation and western blot analysis of TRIM37 and PTEN in PC cells. **(C, D)** Western blot analysis of PTEN **(C)** in PC cells. **(D)** Western blot analysis of PTEN in PC cells after incubation with 20 μM MG132 for the indicated times. **(E)** Western blot analysis of PTEN in PC cells treated with 35 μg/ml cycloheximide for the indicated times. **(F)** Immunoprecipitation of PTEN and western blot analysis of HA in PC cells overexpressing TRIM37 and HA-ubiquitin.

The specific interaction between TRIM37 and PTEN and their co-localization in PANC-1 and MIA PaCa-2 cells were confirmed by immunofluorescent staining and co-immunoprecipitation assays (**Figures 5A, B**). Moreover, western blot analysis showed that PTEN levels were decreased in cells overexpressing TRIM37 and elevated in TRIM37-silenced cells (**Figure 5C**), suggesting that TRIM37 might directly promote PTEN degradation. To determine whether TRIM37-mediated ubiquitination promoted proteasomal degradation of PTEN, PC cells overexpressing TRIM37 were incubated with MG132, a proteasome inhibitor. Importantly, this analysis demonstrated that exposure to MG132 upregulated PTEN expression in both control (empty vector) and TRIM37-overexpressing PC cells (**Figure 5D**). Consistent with this, the half-life of PTEN protein was shorter in TRIM37-overexpressing PC cells compared with control cells (**Figure 5E**). To test whether TRIM37-induced PTEN downregulated through the ubiquitin-proteasome pathway, we performed ubiquitination assays by co-transfecting PC cells with HA-tagged ubiquitin and a FLAG-TRIM37 overexpression vector followed by immunoprecipitation and western blot analysis. We found that cells overexpressing TRIM37 contained significantly more polyubiquitinated PTEN compared with control cells, whereas cells with TRIM37 silencing contained significantly less (**Figure 5F**). These data demonstrate that TRIM37 functions as an E3 ubiquitin ligase in PC cells and targets PTEN for ubiquitination and proteasomal degradation.

### TRIM37 Promotes PC 5-FU Resistance Through Inhibition of PTEN

**Figure 6:**
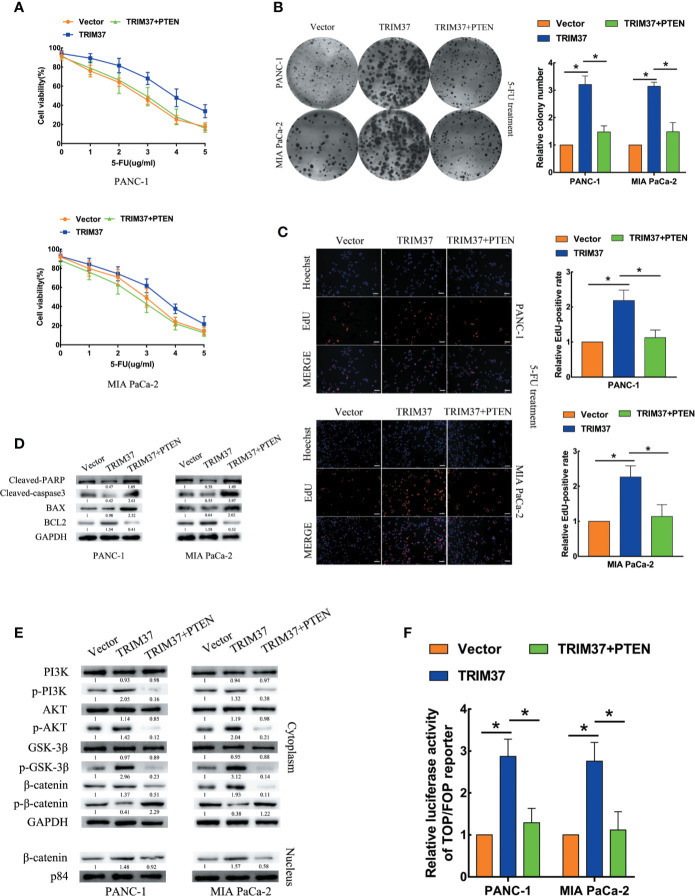
TRIM37 promotes 5-FU resistance of PC cells through PTEN inhibition. **(A–C)** CCK-8 **(A)**, colony formation **(B)**, and EdU, scale bar, 100 um **(C)** assays of the indicated cells overexpressing PTEN after incubation with 5-FU. **(D)** Western blot analysis of cleaved PARP, cleaved caspase-3, BAX and BCL2 in the indicated cells. **(E)** Western blot analysis of PI3K, p-PI3K, AKT, p-AKT, GSK-3β, p-GSK-3β, β-catenin, p-β-catenin, and nuclear β-catenin in the indicated cells. **(F)** TCF4/LEF1-driven luciferase reporter assay of the indicated cells. *(P < 0.05).

Next, we determined whether PTEN degradation was required for TRIM37-mediated AKT–GSK-3β–β-catenin pathway activation and promotion of 5-FU resistance in PC cells. To this end, we examined the ability of PTEN overexpression to reverse the effects of elevated TRIM37 expression. Indeed, cell viability, colony formation, and proliferation assays indicated that TRIM37-induced inhibition of resistance to 5-FU was mediated through inhibition of PTEN (**Figures 6A–C**). PTEN overexpression partly inhibited the apoptosis mediated by 5-FU resistance in TRIM37 overexpressing PC cells, as illustrated by elevated expression of cleaved PARP, cleaved caspase-3, and BAX, but decreased expression of BCL2 (**Figure 6D**), as well as decreased expression of p-PI3K, p-AKT, p-GSK-3β, and cytoplasmic/nuclear β-catenin in western blot analysis (**Figure 6E**). Furthermore, PTEN overexpression significantly inhibited the ability of TRIM37 overexpression to promote TCF4/LEF1-driven luciferase activity (**Figure 6F**).

### TRIM37 and PTEN Expression in PC Correlates With Patient Prognosis

**Figure 7:**
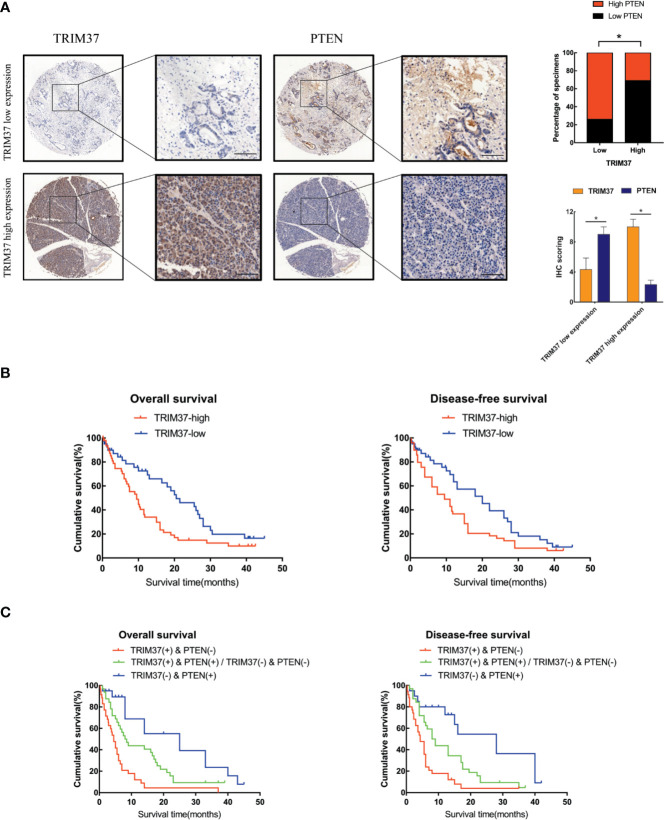
High TRIM37 and low PTEN expression predicts worse prognosis for PC patients. **(A)** IHC staining of TRIM37 and PTEN in representative clinical PC specimens with low TRIM37/high PTEN expression (top) and high TRIM37/low PTEN expression (bottom), scale bar, 100 um. **(B, C)** Kaplan–Meier survival analysis of 110 PC patients stratified by TRIM37 and PTEN expression levels indicates that high TRIM37 and low PTEN expression correlates with poor overall survival and disease-free survival in PC patients. *(P < 0.05).

Lastly, we analyzed the relationship between survival of PC patients and TRIM37 and PTEN expression in 110 cases human PC specimens. This analysis revealed that TRIM37 and PTEN expression were significantly negatively correlated in PC tissues (**Figure 7A**, **Supplementary Table 1**), consistent with our findings *in vitro*. Survival analysis indicated that patients with high TRIM37 levels combined with low PTEN levels had a worse prognosis than patients with low TRIM37 and high PTEN levels (**Figures 7B, C**).

## Discussion

The majority of PC-related deaths can be attributed to chemoresistance and/or recurrence, but little is known of the molecular mechanisms underlying either of these processes. A clearer understanding of the molecules involved and their regulation would therefore help in the design of therapeutic strategies to overcome chemoresistance and prevent recurrence. In a previous study, we showed that TRIM37 was frequently upregulated in PC and that its overexpression was positively associated with tumor growth and worse patient prognosis, leading us to hypothesize that elevated TRIM37 expression may play an important role in PC development and progression. Here, we employed *in vitro* and *in vivo* approaches to examine the role of TRIM37 in PC. We showed not only that TRIM37 contributes to CSC-like traits of PC cells but also that it mediates 5-FU resistance by modulating PTEN degradation and activation of the AKT–GSK-3β–β-catenin pathway.

Although TRIM family proteins are known to play a role in many cancers, the importance of their E3 ubiquitin ligase activity in the initiation and development of cancer is poorly understood. TRIM67 has been shown to play a tumor suppressor role by activating wild-type P53 and modulating chemoresistance in colorectal cancer (30). TRIM65 is a functional target of the microRNA miR-138-5p, which regulates autophagy and cisplatin resistance in the none small-cell cancer cell line A549/DDP (31). TRIM proteins are also considered to be crucial regulators of cancer cell migration and invasiveness by promoting the ubiquitination and degradation of binding partners. Tan and colleagues showed that TRIM59 stabilizes the apoptosis-related protein PDCD10 by inhibiting its ubiquitination and subsequent P62-selective autophagic degradation; in turn, this promoted RhoA and ROCK1 activation and metastasis of breast cancer cells (32). Wei et al. demonstrated that TRIM65 enhances the invasiveness of urothelial carcinoma of the bladder *via* the ubiquitination and degradation of ANXA2 and modulation of cytoskeleton rearrangement and epithelial–mesenchymal transition (33). Thus, the E3 ligase function of TRIM family proteins appears to correlate well with their ability to drive the initiation, chemoresistance, and metastasis of multiple cancers. Further in-depth investigation of the molecular mechanisms involved will help to clarify the role of TRIM family proteins in tumor progression.

Previous studies suggested the possibility that TRIM37 may have essential functions in various cancer types. For example, TRIM37 is overexpressed in breast cancers and promotes tumor progression by acting as an oncogenic H2A ubiquitin ligase and suppressing the activity of several tumor suppressor proteins (15). We previously showed that TRIM37 is an oncogene that promotes PC and hepatocellular carcinoma *via* β-catenin and PI3K–AKT signaling (15). In the current study, we extended these findings by demonstrating that TRIM37 activates AKT–GSK-3β–β-catenin signaling *via* ubiquitination and degradation of PTEN. Wu et al. found that TRIM37 plays a vital role in genotoxic activation of NF-κB *via* monoubiquitination of nuclear NEMO at lysine 309, resulting in its nuclear export and activation of IKK–NF-κB in esophageal cancer (34). To verify the E3 ligase-mediated function of TRIM37, we isolated TRIM37 protein complexes and identified the binding proteins by IP-MS. This analysis confirmed that TRIM37 binds to and ubiquitinates PTEN and promotes its proteasomal degradation in PC cells.

PTEN is mutated in numerous cancers and has been extensively investigated for its role as a tumor suppressor by negative regulation of AKT signaling in multiple cancers (35–38). Here, we identified an inverse relationship between the expression of TRIM37 and PTEN in clinical PC specimens, which is consistent with TRIM37-mediated regulation of PTEN degradation. Moreover, in keeping with the role of PTEN as a tumor suppressor, we found that high TRIM37 and low PTEN expression levels were associated with poorer survival in patients with PC, confirming the implications suggested by our *in vitro* mechanistic analyses and mouse xenograft study.

**Figure 8:**
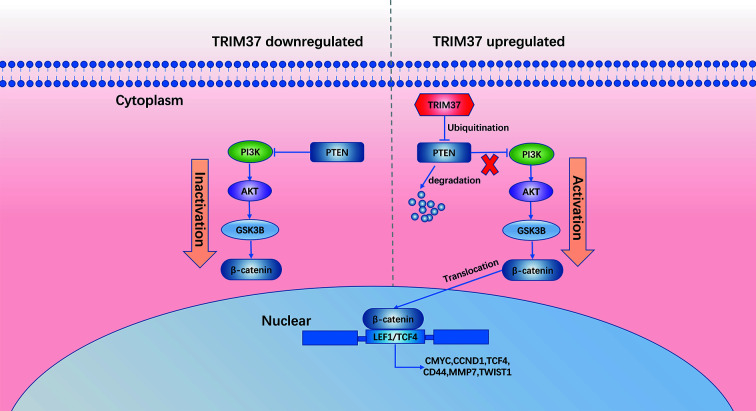
Schematic diagram of our working model in this study.

In summary, our results demonstrate that TRIM37 overexpression in PC may play an essential role in the development of chemoresistance and stemness, leading to poorer prognosis. TRIM37 plays an oncogenic role in PC by opposing the activity of PTEN in regulating the AKT–GSK-3β–β-catenin signaling pathway (**Figure 8**). Therefore, our research suggests that TRIM37 may be a novel target for the early treatment of PC.

## Data Availability Statement

The original contributions presented in the study are included in the article/**Supplementary Material**; further inquiries can be directed to the corresponding authors.

## Ethics Statement

The studies involving human participants were reviewed and approved by the Human Research Ethics Committee at The Affiliated Hospital of Guizhou Medical University. The patients/participants provided their written informed consent to participate in this study. The animal study was reviewed and approved by the Committee on the Ethics of Animal Experimentation of Guizhou Medical University.

## Author Contributions

CS and CY lead this study. SC, ZH, and CZ carried out the experiments. Data analyses were performed by YL and LL. LD and JW assisted in harvesting tissues samples and providing clinical data. All authors contributed to the article and approved the submitted version.

## Funding

This work was supported by the National Natural Science Foundation of China [grant numbers 81672906, 81560477, 81660483, 81860505, 81860506] and Project of Graduate Research Fund of Guizhou Province in 2019 [grant number Qianjiaohe YJSCXJH (2019) 072], grant number Qiankehe Pingtairencai [2016] 5647, Qiankehe Pingtairencai [2017] 5404, Qianshengzhuanhezi [2012] 94, Qianjiaoyanhe GZS [2016] 09.

## Conflict of Interest

The authors declare that the research was conducted in the absence of any commercial or financial relationships that could be construed as a potential conflict of interest.

## Supplementary Material

Supplementary Figure 1 **(A)** Western blot analysis of TRIM37 in the indicated PC cells. **(B)** CCK-8 assay showed the cell viability of PC cells treated with 5-FU under different time point.

Supplementary Figure 2 IHC staining of BMI-1, LGR-5, OCT4A, NANOG, and SOX2 in sections of PANC-1 tumors excised from the mice, scale bar, 100 um.

## Abbreviations

PC, Pancreatic cancer; PTEN, Phosphatase and tensin homolog; CSCs, Cancer stem cells; TRIM, Tripartite motif-containing; RING, Really Interesting New Gene; AKT, Protein kinase B; GSK-3β, Glycogen synthase kinase-3β; PI3K, Phosphatidylinositol 3-kinase; ATCC, American Type Culture Collection; qRT-PCR, Quantitative real-time polymerase chain reaction; DMEM, Dulbecco’s Modified Eagle’s Medium; FBS, Fetal bovine serum; shRNA, short hairpin RNA; IP-MS, Immunoprecipitation and mass spectrometry; 5-FU, 5-Fluorouracil; KEGG, Kyoto Encyclopedia of Genes and Genomes.
