# Supplementary figures and images for: TRIM37 Mediates Chemoresistance and Maintenance of Stemness in Pancreatic Cancer Cells via Ubiquitination of PTEN and Activation of the AKT–GSK-3β–β-Catenin Signaling Pathway
Source: Front Oncol. 2020 Oct 16;10:554787. doi: 10.3389/fonc.2020.554787 (PMC7651862; doi:10.3389/fonc.2020.554787)

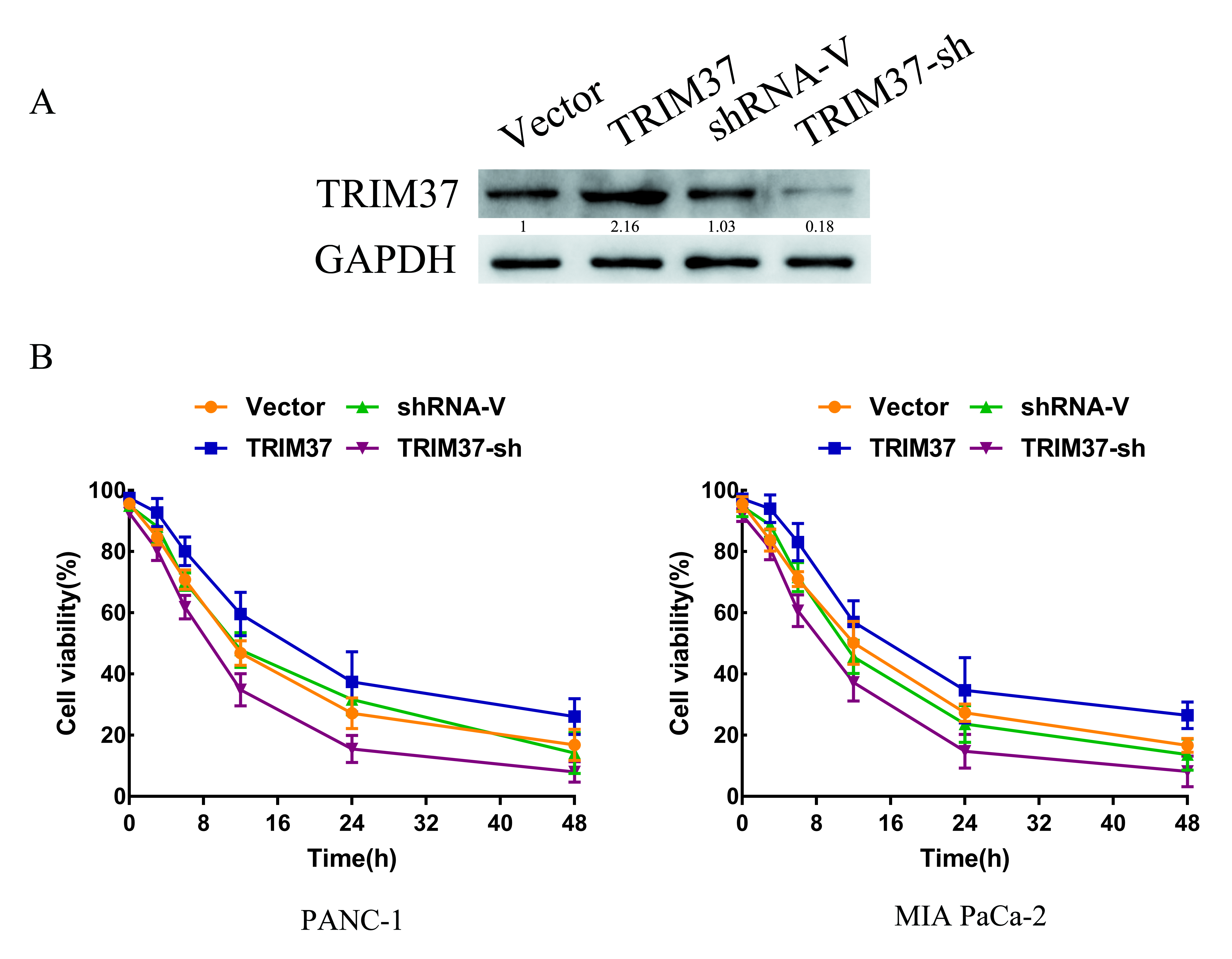

Supplement: Supplementary Figure 1 — (A) Western blot analysis of TRIM37 in the indicated PC cells. (B) CCK-8 assay showed the cell viability of PC cells treated with 5-FU under different time point. [file Image_1.tif]

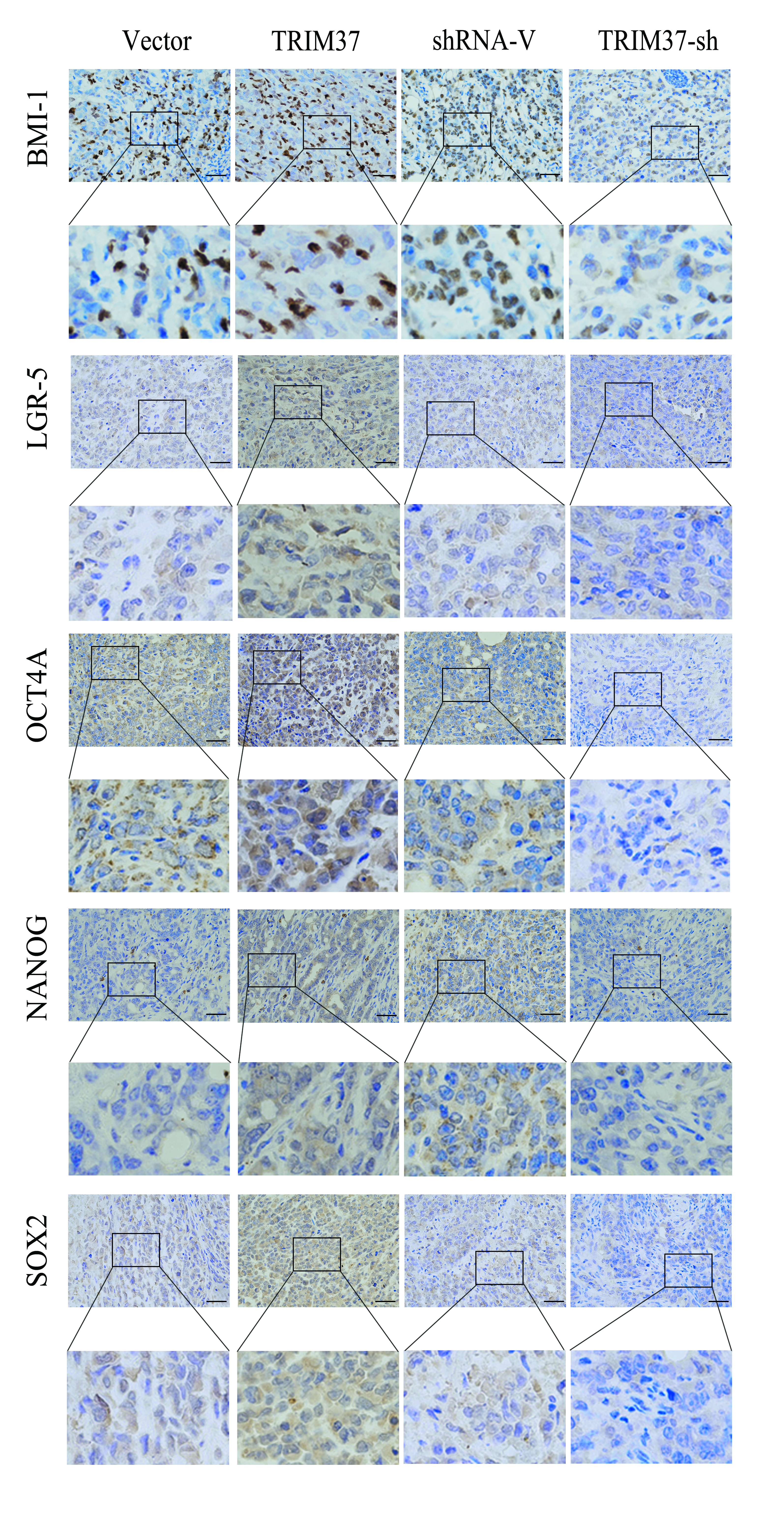

Supplement: Supplementary Figure 2 — IHC staining of BMI-1, LGR-5, OCT4A, NANOG, and SOX2 in sections of PANC-1 tumors excised from the mice, scale bar, 100 um. [file Image_2.tif]
