# Supplementary material for: TRIM37 Mediates Chemoresistance and Maintenance of Stemness in Pancreatic Cancer Cells via Ubiquitination of PTEN and Activation of the AKT–GSK-3β–β-Catenin Signaling Pathway
Source: Front Oncol. 2020 Oct 16;10:554787. doi: 10.3389/fonc.2020.554787 (PMC7651862; doi:10.3389/fonc.2020.554787)
Supplement: Supplementary file 4 [file Table_2.docx]

**qPCR Primers**

BMI-1 (NCBI Gene ID :648)

Forward Primer CGTGTATTGTTCGTTACCTGGA

Reverse Primer TTCAGTAGTGGTCTGGTCTTGT

LGR-5 (NCBI Gene ID :8549)

Forward Primer CACCTCCTACCTAGACCTCAGT

Reverse Primer CGCAAGACGTAACTCCTCCAG

NANOG (NCBI Gene ID :79923)

Forward Primer CCCCAGCCTTTACTCTTCCTA

Reverse Primer CCAGGTTGAATTGTTCCAGGTC

OCT4A (NCBI Gene ID :5460)

Forward Primer CTGGGTTGATCCTCGGACCT

Reverse Primer CCATCGGAGTTGCTCTCCA

SOX2 (NCBI Gene ID :6657)

Forward Primer TACAGCATGTCCTACTCGCAG

Reverse Primer GAGGAAGAGGTAACCACAGGG

GAPDH (NCBI Gene ID :2597)

Forward Primer ACAACTTTGGTATCGTGGAAGG

Reverse Primer GCCATCACGCCACAGTTTC

MYC (NCBI Gene ID :4609)

Forward Primer TCCCTCCACTCGGAAGGAC

Reverse Primer CTGGTGCATTTTCGGTTGTTG

CCND1 (NCBI Gene ID :595)

Forward Primer TGGAGCCCGTGAAAAAGAGC

Reverse Primer TCTCCTTCATCTTAGAGGCCAC

TCF4 (NCBI Gene ID :6925)

Forward Primer GGCTATGCAGGAATGTTGGG

Reverse Primer GTTCATGTGGATGCAGGCTAC

MMP7 (NCBI Gene ID :4316)

Forward Primer GAGTGAGCTACAGTGGGAACA

Reverse Primer CTATGACGCGGGAGTTTAACAT

TWIST1 (NCBI Gene ID :7291)

Forward Primer GTCCGCAGTCTTACGAGGAG

Reverse Primer GCTTGAGGGTCTGAATCTTGCT

CD44 (NCBI Gene ID :960)

Forward Primer CTGCCGCTTTGCAGGTGTA

Reverse Primer CATTGTGGGCAAGGTGCTATT
