# Supplementary material for: TRIM37 Mediates Chemoresistance and Maintenance of Stemness in Pancreatic Cancer Cells via Ubiquitination of PTEN and Activation of the AKT–GSK-3β–β-Catenin Signaling Pathway
Source: Front Oncol. 2020 Oct 16;10:554787. doi: 10.3389/fonc.2020.554787 (PMC7651862; doi:10.3389/fonc.2020.554787)
Supplement: Supplementary file 5 [file Table_3.docx]

| **No.** | **Name** | **Company** | **Catalog number** | **Dilution ratio** | **Molecular weight（kDa）** |
| --- | --- | --- | --- | --- | --- |
| 1 | BMI-1 | CST | #6964 | WB: 1：1000  IHC: 1:200 | 41-43 |
| 2 | LGR-5 | abcam | ab75732 | WB: 1:1000 - 1:2000  IHC: 1:10 -1:50 | 100 |
| 3 | NANOG | CST | #4903 | WB: 1：2000  IHC: 1:800 | 42 |
| 4 | OCT4A | CST | #2890 | WB: 1：1000  IHC: 1:1200 | 45 |
| 5 | SOX2 | CST | #14962 | WB: 1：1000  IHC: 1:300 | 35 |
| 6 | GAPDH | CST | #5174 | WB: 1：1000 | 37 |
| 7 | Cleaved-PARP | CST | #5625 | WB: 1：1000 | 89 |
| 8 | Cleaved-caspase3 | abcam | ab2302 | WB: 1：1000 | 17 |
| 9 | BAX | CST | #5023 | WB: 1：1000 | 20 |
| 10 | BCL2 | CST | #15071 | WB: 1：1000 | 26 |
| 11 | PI3K | CST | #4255 | WB: 1：1000 | 110 |
| 12 | p-PI3K | abcam | ab138364 | WB: 1:500 - 1:1000 | 81 |
| 13 | AKT | CST | #2920 | WB: 1:2000 | 60 |
| 14 | p-AKT | abcam | ab81283 | WB: 1:5000 - 1:10000  IHC: 1:100 -1:250 | 56 |
| 15 | GSK-3β | CST | #12456 | WB: 1：1000 | 46 |
| 16 | p-GSK-3β | CST | #9323 | WB: 1：1000  IHC: 1:50 | 46 |
| 17 | β-catenin | CST | #8480 | WB: 1：1000  IHC: 1:100 | 92 |
| 18 | p-β-catenin | abcam | ab81305 | WB: 1:10000- 1:20000 | 70-86 |
| 19 | p84 | abcam | ab487 | WB: 1:500 - 1:2000 | 84 |
| 20 | MYC | CST | #9402 | WB: 1：1000 | 57-70 |
| 21 | CCND1 | CST | #2922 | WB: 1：1000 | 36 |
| 22 | TCF4 | abcam | ab185736 | WB: 1:500 - 1:2000 | 71 |
| 23 | MMP7 | abcam | ab5706 | WB: 1:500 - 1:2000 | 28 |
| 24 | TWIST1 | CST | #46702 | WB: 1：1000 | 26 |
| 25 | CD44 | CST | #3570 | WB: 1：1000 | 80 |
| 26 | TRIM37 | abcam | ab264190  ab238156 | WB: 1:2000 - 1:10000  IP: 2 - 5 µg/ml  IHC: 1:20 -1:200  IF: 1:50 -1:200 | 107 |
| 27 | PTEN | CST  abcam | #9188  ab32199 | WB: 1：1000  IF: 1:150 - 1:500  IP: 1:50  IHC: 1:125 | 54 |
| 28 | HA | CST | #3724 | WB: 1：1000 | 1 |
| 29 | PCNA | CST | #13110 | IHC: 1:8000 | 36 |
| 30 | Ki-67 | CST | #9449 | IHC: 1:400 | - |
